# Analysis of Vessel Segmentation Based on Various Enhancement Techniques for Improvement of Vessel Intensity Profile

**DOI:** 10.1155/2022/7086632

**Published:** 2022-06-28

**Authors:** Sonali Dash, Sahil Verma, Kavita, SeongKi Kim, Jana Shafi, Muhammad Fazal Ijaz

**Affiliations:** ^1^Department of Computer Science and Engineering, Chandigarh University, Mohali 140413, India; ^2^National Centre of Excellence in Software, Sangmyung University, Seoul, Republic of Korea; ^3^Department of Computer Science, College of Arts and Science, Prince Sattam bin Abdul Aziz University, Wadi Ad-Dawasir 11991, Saudi Arabia; ^4^Department of Intelligent Mechatronics Engineering, Sejong University, Seoul 05006, Republic of Korea

## Abstract

It is vital to develop an appropriate prediction model and link carefully to measurable events such as clinical parameters and patient outcomes to analyze the severity of the disease. Timely identifying retinal diseases is becoming more vital to prevent blindness among young and adults. Investigation of blood vessels delivers preliminary information on the existence and treatment of glaucoma, retinopathy, and so on. During the analysis of diabetic retinopathy, one of the essential steps is to extract the retinal blood vessel accurately. This study presents an improved Gabor filter through various enhancement approaches. The degraded images with the enhancement of certain features can simplify image interpretation both for a human observer and for machine recognition. Thus, in this work, few enhancement approaches such as Gamma corrected adaptively with distributed weight (GCADW), joint equalization of histogram (JEH), homomorphic filter, unsharp masking filter, adaptive unsharp masking filter, and particle swarm optimization (PSO) based unsharp masking filter are taken into consideration. In this paper, an effort has been made to improve the performance of the Gabor filter by combining it with different enhancement methods and to enhance the detection of blood vessels. The performance of all the suggested approaches is assessed on publicly available databases such as DRIVE and CHASE_DB1. The results of all the integrated enhanced techniques are analyzed, discussed, and compared. The best result is delivered by PSO unsharp masking filter combined with the Gabor filter with an accuracy of 0.9593 for the DRIVE database and 0.9685 for the CHASE_DB1 database. The results illustrate the robustness of the recommended model in automatic blood vessel segmentation that makes it possible to be a clinical support decision tool in diabetic retinopathy diagnosis.

## 1. Introduction

In the healthcare industry, biomedical images are the primary data source and, simultaneously, utmost hard for analysis. Artificial intelligence (AI) is a technique to automatically analyze and prevent the elevated risks of developing chronic conditions and help patients avoid long-term health problems. As these automated structures become widespread in the healthcare industry, they may bring about progressive changes for radiologists, clinicians, ophthalmologists, and even patients using imaging technology to monitor the treatments. Thus, it is vital to develop an appropriate prediction model and link carefully for measurable events such as clinical parameters and patient outcomes to analyze the severity of the disease. This permits clinicians to obtain warnings about potential measures before they occur, making more choices about how to progress with a decision to prevent the disease's progression.

The retinal blood vessel is the only part that may catch a straight noninvasively in vivo. Blood vessel plays a significant role in automatic identification as it contains the issue of screening systems. Accurate segmentation and vessel length analysis, orientation, and thickness can make clear the assessment of retinopathy of prematurity, identification of reduction arteriolar, and evaluation of vessel width for the recognition of ailments such as diabetes, arteriosclerosis, hypertension, and so on [1–3]. We can read about many ideas to improve the blood vessel segmentation by computing the contrast of retinal blood vessels and background. Computerized evaluation of vasculatures has been extensively recognized as the initial stage in the progress of a computer-aided investigative scheme for ocular ailments. Several suggested rules have been recommended for vessel segmentation [4]. Few commonly suggested algorithms for vessel extraction are discussed. Few authors have proposed a vessel segmentation process using a matched filter. Images at different scales are convolved with the filter, and the highest output is noted at each pixel [5–7]. With an assumption of elongated vessels, Staal et al. presented an idea of ridge-based vessel segmentation in which image ridges are transformed to control line elements [8]. It has recommended an adaptive local multi-threshold probing algorithm. For multithreshold probing, they have used different thresholds in series for computation [8]. In the literature, it has introduced automatic vessel tree segmentation by combining shifted filter responses (COSFIRE) [9, 10]. The literature suggests using BCOSFIRE and generalized matrix learning vector quantization (GMLVG) to detect the blood vessel [11]. Mapayi et al. have discussed and compared vessel segmentation based on global thresholding [12]. Many filters are introduced by various researchers for retinal blood vessel segmentation, such as median, Gaussian, matched, Gabor, Cake, steerable, Frangi, and many more [5, 13–19].

Afterwards, many extensions of the existing techniques in several directions are recommended for blood vessel segmentation by various researchers [20–22]. Few of the various extended filters are discussed. The original median filter is extended as an improved median filter (IMF), hybrid median filter (HMF), and weighted median filter (WMF) for vessel segmentation [23]. Several expansions of matched filter (MF) are utilized; for example, MF is integrated with pulse coupled neural networks, and the Otsu algorithm is applied for segmentation [24]. It has been suggested in the literature to improve the matched filter through the ant colony algorithm and through Clifford matched filter [25, 26]. A zero mean Gaussian matched filter is introduced based on a first-order derivative of the Gaussian filter [27]. An upgraded version of the matched filter is suggested through an optimization technique [28, 29]. The matched filter is upgraded through another optimization technique, that is, genetic algorithm [30]. Another recommended way to improve the matched filter is using particle swarm optimization [31, 32].

Correspondingly, for the Gabor filter, there are many expansions presented in the literature. A multi-scale, multi-directional Gabor wavelet transform and created feature vector consisting of pixel intensity and maximum response achieved for Gabor filter at various scales are recommended. Afterward, they utilized a classification algorithm known as linear minimum squared error (LMSE) [33]. Two-dimensional Gabor wavelet with a Gaussian mixture model is presented to classify a pixel as a vessel or a nonvessel [34]. Two different approaches are compared for blood vessel extraction. In the first approach, they have employed Gaussian filtering for preprocessing, LoG filtering to enhance the retinal image, and adaptive thresholding for the segmentation task. In the second approach, they have utilized unsharp masking for preprocessing, Gabor wavelet to enhance the retinal image, and global thresholding for the segmentation task [35]. A technique is suggested for the green channel noise reduction of the retina by employing a low pass radius filter and followed by the Gabor filter and a Gaussian fractional derivative for enhancement of blood vessels [36]. Gabor filter is extended by integrating Gabor, Frangi, and Gaussian filters with top-hat transform [37]. A new technique is introduced to design a set of 180 Gabor filters with variable scales and elongated variables by applying an optimization approach known as competitive imperialism algorithm (CIA) for vessel segmentation [38]. A new hybrid scheme is suggested by combining the existing techniques in which multi-scale vessel enhancement (MSVE), morphological operations, bottom-hat transform, and image fusion are combined for blood vessel extraction [36]. Gabor filter and Hessian method are used together for enhancing the features. Then K-mean clustering is utilized for vessel extraction [39]. A new improved curvelet transform technique is suggested to detect thick and thin blood vessels for extraction [40]. A hybrid method by combining two different existing techniques such as lateral inhibition and differential evolution is used for vessel segmentation [41]. Existing supervised and unsupervised machine learning techniques are utilized for vessel segmentation by employing image features [42]. To enhance the performance of the original Frangi filter, it is combined with the existing probabilistic patch-based denoiser for vessel segmentation [43].

Newly deep learning that is a supervised approach has been effectively employed for biomedical image processing that includes retinal blood vessel segmentation. Wang et al. have suggested context spatial U-Net for the segmentation of blood vessels [44, 45]. Chen et al. have discussed many deep learning approaches for vessel segmentation in their review paper, where better results are achieved [46]. Many machine learning algorithms are available in the literature for various disease detections [47–52]. However, deep learning applications depend on an enormously huge database. Moreover, annotated data sets are not readily available compared to other imaging fields. Annotation of medical data is a costly, complicated, and lingering process and thus experts need more time.

Additionally, an annotation may not always be possible for rare health issues. Consequently, the availability of medical data is a significant obstacle for deep learning approaches. Although deep learning methods have achieved substantial achievement, decent theory for deep learning algorithms is still absent. Models of deep learning offer good results, and the researchers are utilizing continuously deprived of having an understandable knowledge of attaining higher results and the work process. Another critical challenge is the legal association of black box utility. It can be a barrier because healthcare experts would not depend on it. If the results achieved are wrong, then who could be accountable. Because of this sensitive issue, hospitals may not be convenient with the black box, that is, how it could draw that particular result from the ophthalmologist.

Therefore, understanding deep learning techniques and their hidden layers working for a given problem is a great challenge for researchers. Furthermore, in the event of the source of data changes, the problem occurs in network response, which most researchers do not address. That will be the influence of modification in a data acquirement device because this may give on to variations in features of images like colour intensity levels or illumination. Thus, the absence of generalize ability will harm the performance of deep learning networks. Accordingly, it is concluded that deep learning networks still deliver higher performance results depending on huge image databases. Consequently, it needs large storage and memory with excess training time for the networks. The insufficient availability of large biomedical imaging data sets is another hurdle in developing a deep learning network [53, 54].

Consequently, to enhance the performance of any model whether supervised or unsupervised, the quality of the image has a great impact on the performance model. Few factors in the image like uneven illumination or camera position can affect the image contrast, resulting in inadequate features in the image. Thus, image enhancement is a very important part of preprocessing and the proper selection of enhancement techniques can improve the effectiveness of the existing models to a great extent. As a consequence, it is essential to research the relationship between image enhancement and the existing models. Thus, in this work, an unsupervised approach, that is, traditional Gabor filter, is chosen to improve its performance by employing various enhancement techniques.

Six different enhancement algorithms are used in the proposed work. The advantages and disadvantages of a particular enhancement algorithm are difficult to describe because of the reliable and consistent measures for the evaluation of the superiority of the enhanced image. Thus, based on the experimental results, the best-integrated model is derived.

After an extensive study of the literature, it is noted that many existing techniques are taken into consideration for modifying and improving their performances. Therefore, existing methods can still be considered for fundus image segmentation by upgrading them and boosting their computational ability. Gabor filters are found to be effectively suitable in the segmentation of retinal images because of oriented features as the vessels of the retina are linked and piecewise linear [34]. Furthermore, Gabor filters can be adjusted to particular frequencies and thus can be adjusted to enhance the blood vessel. Although from the literature it is observed that there are many techniques available for vessel extraction utilizing various filters and enhancement methods, still, a lot can be done to ameliorate further. Designing a particular enhancement technique is infeasible, as it generates a visual artifact-free output. Selecting a specific enhancement scheme is hard since parameters in assessing output quality are not available. Furthermore, usually enhancement algorithms rely on authentic parameter selection. This prompted a recommendation a robust enhanced Gabor filter by integrating it with various enhanced techniques such as gamma corrected adaptively with distributed weights (GCADW) [55, 56], homomorphic filter [57, 58], joint equalization of histogram (JEH) [59, 60], unsharp masking filter, adaptive unsharp masking filter [61, 62], and particle swarm optimization (PSO) based unsharp masking filter [63, 64].

Additionally, it is noted in the literature survey that the researchers have combined two to three existing techniques for the improvement of original approaches. In this work, we have presented an idea to improve blood vessel segmentation through illumination-robust Gabor filter by combining it with six enhancement techniques. The main contributions of the suggested approach are covered in few steps as follows:

- Initially, the existing Gabor filter is used to enhance the fundus image, and hysteresis thresholding is applied for vessel segmentation.
- In the second step, different enhancement techniques are combined individually with the Gabor filter to make its illumination robust and to improve its performance followed by hysteresis thresholding for vessel segmentation.
- In the final postprocessing step, a morphological cleaning operation is performed to clean undesired pixels that may lead to more false positives.

The suggested methods are assessed on DRIVE and CHASE_DB1data sets, and based on the results, the best-integrated model is finalized.

Table 1 shows the summary of the advantages and disadvantages of various vessel segmentation methods.

## 2. Preliminary Concepts

### 2.1. Gabor Filter

Gabor filters are influential techniques that have been extensively utilized for multi-scale and multi-directional analysis in image processing. Because of its directional selectiveness ability to detect oriented features, it is extended by proposed fine-tuning. As a result, precise frequencies and scale are shown in filter performance as low-level oriented edge discriminators. The features from the Gabor filter can be extracted from the original image as described below [34]:

(1) $$\begin{array}{c} G\left(x,y\right)=f\left(x,y\right)⊗P_{f}\left(x,y\right), \end{array}$$

where *f*(*x*, *y*) is the original image and *P*_*f*_ (*x*, *y*) is the impulse response of the 2-D Gabor filter. The symbol ⊗ represents the convolution sum.

### 2.2. Gamma Corrected Adaptively with Distributed Weights (GCADW)

GCADW utilizes cumulative distribution function (cdf) and employed normalized gamma function to it. They have achieved a modified transformation curve where histogram statistics are available. Accordingly, substantial adjustment can be done in the lower gamma parameter. Thus, they have formulated adaptive gamma correction (AGC) to process intensity in consecutive increments of the original trend. AGC is defined as follows:

(2) $$\begin{array}{c} T\left(l\right)=l_{\max}{\left(\frac{l}{l_{\max}}\right)}^{\gamma }=l_{\max}{\left(\frac{l}{l_{\max}}\right)}^{1-cdf\left(l\right)}, \end{array}$$

where *l* is the intensity of the input image, *l*_max_ is the maximum intensity of the input and *γ* is the varying adaptive parameter. The low intensity can be increased substantially without decreasing the high intensity by applying AGC technique. Additionally, weighting distribution (WD) function applied is also employed for the modification of statistical histogram to some extent for the reduction of adverse effect. The WD function is defined as follows:

(3) $$\begin{array}{c} pdf_{w}\left(l\right)=pdf_{\max}{\left(\frac{pdf\left(l\right)-pdf_{\min}}{pdf_{\max}-pdf_{\min}}\right)}^{a}, \end{array}$$

where *a* is an adjustable parameter, pdf_max_ is the probability density function with a maximum value of the statistical histogram, and pdf_min_ is the probability density function with minimum value. Considering the (3), the revised cdf is approached as follows:

(4) $$\begin{array}{c} cdf_{w}\left(l\right)=\frac{\sum_{l=0}^{l_{\max}}pdf_{w}\left(l\right)}{\sum^{}pdf_{w}}, \end{array}$$

where the sum *pdf*_*w*_ is computed as follows:

(5) $$\begin{array}{c} \sum^{}pdf_{w}=\overset{l_{\max}}{\underset{l}{\sum }}pdf_{w}\left(l\right). \end{array}$$

In conclusion, the value of parameter gamma derived from cdf equation (3) is altered as follows:

(6) $$\begin{array}{c} \gamma =1-cdf_{w}\left(l\right). \end{array}$$

### 2.3. Homomorphic Filter

Many suggested approaches are available to enhance images utilizing a homomorphic filter [57]. Information missing in dark regions can be identified by equalizing the light variations onto the image. An image can be denoted as a product of two components as seen in the following equation:

(7) $$\begin{array}{c} I\left(x,y\right)=L\left(x,y\right)*R\left(x,y\right), \end{array}$$

where *L* (*x*, *y*) is the illumination and *R* (*x*, *y*) is the reflectance components of the original image. The filter function for the homomorphic filter chosen is as follows:

(8) $$\begin{array}{c} H\left(u,\;v\right)=\;\left(\gamma_{h}-\gamma_{l}\right)\left[1-\text{exp}\left\{k{\left(\frac{P\left(u,v\right)}{P_{0}}\right)}^{2}\right\}\right]+\gamma_{l}, \end{array}$$

where *k* controls the steepness and is taken as constant, *P*_0_ is the frequency of cut-off value, the measured distance of the origin Fourier transform is represented as *P*(*u*, *v*), and *γ*_*l*_, *γ*_*h*_ are the low- and high-frequency gain, respectively.

### 2.4. Joint Equalization of Histogram

Joint histogram equalization is an approach where modification of histograms and enhancement of contrast in digital images are implemented [59]. The entire joint histogram equalization process is explained below.

By using a neighbouring window of *Z*^2^, the gray value pixel *g*(*p*, *q*) is calculated and defined below:

(9) $$\begin{array}{c} g\left(p,q\right)=\frac{1}{z\times z}\overset{k}{\underset{m=-k}{\sum }}\overset{k}{\underset{n=-k}{\sum }}f\left(p+m,q+n\right). \end{array}$$

The joint histogram is as follows:

(10) $$\begin{array}{c} H=\left\{h\left(a,b\right)|0\leq a\leq C-1,0\leq b\leq C-1\right\}, \end{array}$$

where the expression *h*(*a*, *b*) represents the existence of the gray level pair numbers *f*(*p*, *q*) and *g*(*p*, *q*) around the correspondent spatial location (*p*, *q*) of the images *I* and $\bar{I}$ correspondingly. It signifies the count function. Because *a* and *b* is considered whatever conceivable numeral value among 0 and *C* − 1, the number of pixel pair groupings feasible are *C* × *C*. Thus, the joint histogram H will comprise *C* × *C* entries.

By utilizing the count function, the cumulative distribution function can be achieved as follows:

(11) $$\begin{array}{c} CDF\left(p,q\right)=\overset{i}{\underset{m=0}{\sum }}\overset{j}{\underset{n=0}{\sum }}h\left(m,n\right). \end{array}$$

Two-dimensional CDF value is utilized to produce the output pixel intensity enhanced in contrast. The equalized value of the intensity pairs (*p* and *q*) in the output image can be achieved through the histogram equalization method as follows:

(12) $$\begin{array}{c} h_{eq}\left(p,q\right)=\text{round}\left(\frac{L-1}{MN-1}\left(CDF\left(p,q\right)-CDF\;{\left(p,q\right)}_{\min}\right)\right). \end{array}$$

### 2.5. Unsharp Masking Filter

Local contrast enhancement can be done using unsharp masking. This technique creates a mask of the original image utilizing a negative image. Afterward, the original positive image is combined with the unsharp mask to produce an image that is less blurry than the original. Usually, a linear or nonlinear filter that magnifies the high-frequency components of a signal is said to be an unsharp masking filter.

### 2.6. Adaptive Unsharp Masking Filter

Lin et al. have suggested an adaptive unsharp masking filter to enhance the colour images [61]. They have improved the colour brightness by stretching the colour channel and enhanced the contrast by expanding the edge. Especially, a hyperbolic-tangent function scale is established that regulates the gain, and sharpness is enhanced. The model relates to the intensity of the original image and recognized edges.

Input pixel magnitude and image coordinate edge are the two components of the hyperbolic scheme.

The gain factor considered for the input-based component is defined as follows:

(13) $$\begin{array}{c} \lambda_{g_{pq}}=0.5\left(1+\tanh\left(3-12\times \left|g_{uv}-0.5\right|\right)\right), \end{array}$$

where *p* = 1,…, *P* and *q* = 1,…, *Q* represent width and height of image, respectively, and *λ*_*g*_*pq*__ gain of pixel of I channel, as the RGB image is initially converted to HSI image. While *g*_*pq*_=1/3(*R*+*G*+*B*)_*pq*_ relates to the I channel of HSI colour space, where *R*, *G*, and *B* correspond to the red, green, and blue components of the colour image, respectively.

Thus, to gain adjustment on the detected edge, the subsequent scheme is applied.

(14) $$\begin{array}{c} \lambda_{d_{pq}}=0.5\left(1+\tanh\left(3-6\times \left|d_{uv}\right|-0.5\right)\right), \end{array}$$

where *λ*_*d*_*pq*__ is gain factor defining strength of reconstructed edge *d*_*pq*_.

By multiplying the above two schemes, the complete gain adjustment scheme is obtained and described as follows:

(15) $$\begin{array}{c} \lambda_{pq}=\lambda_{g_{pq}}\lambda_{d_{pq}}. \end{array}$$

Additionally, the measurement of sharpness is evaluated. It is computed from the measurement of the neighbourhood pixel gradient as described below:

(16) $$\begin{array}{c} {\text{Ğ}}_{pq}=\sqrt{\Delta x_{pq}^{2}+\Delta y_{pq}^{2}}, \end{array}$$

(17) $$\begin{array}{c} G=\frac{1}{N}\sum^{}{\text{Ğ}}_{pq},\;\forall pq, \end{array}$$

where Δ*x*_*pq*_=*g*_*pq*_ − *g*_*p*+1,*q*_ and Δ*y*_*pq*_=*g*_*pq*_ − *g*_*p*,*q*+1_ represent the horizontal and vertical gradients across the image, respectively, and *N* is the total number of pixels.

Additionally, an image is evaluated by its colourfulness [65]. Capturing an object under uneven lighting conditions may deteriorate the measurement. The colourfulness is given as below:

(18) $$\begin{array}{c} ∁=\sigma_{RGYB}+0.3\times \mu_{RGYB}, \end{array}$$

where

(19) $$\begin{array}{c} \sigma_{RGYB}=\sqrt{\sigma_{RG}^{2}+\sigma_{YB}^{2}}, \end{array}$$

(20) $$\begin{array}{c} \mu_{RGYB}=\sqrt{\mu_{RG}^{2}+\mu_{YB}^{2}}. \end{array}$$

The colour is computed with respect to the standard deviations *σ*_*rg*_ and *σ*_*yb*_ of colour differences Δ_*rg*_ and Δ_*yb*_, respectively, where

(21) $$\begin{array}{c} \Delta_{RG}=R-G, \end{array}$$

(22) $$\begin{array}{c} \Delta_{YB}=0.5\left(R+G\right)-B, \end{array}$$

where *μ*_*RG*_ and *μ*_*YB*_ signify the means of Δ_*RG*_ and Δ_*YB*_, respectively.

### 2.7. Particle Swarm Optimization (PSO) Based Unsharp Masking Filter

Particle swarm optimization (PSO) is implemented to design the kernel and gain on an unsharp masking filter [63]. The authors have applied a symmetrical kernel through PSO for extraction of the edge with an optimum scale factor for the enhancement of colour image. PSO is utilized to achieve filter kernel settings and after maximizing the information's content to minimize high ranged pixels.

## 3. Proposed Methodology

**Figure 1:**
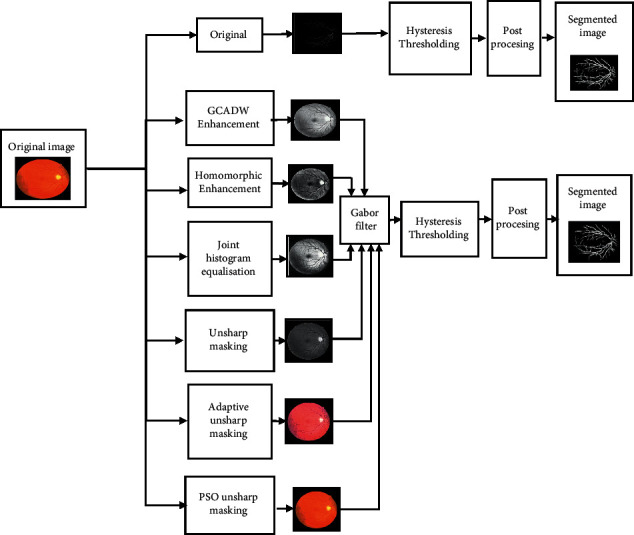
Block diagram of the recommended methods.

The main aim of the proposed approach is to enhance retinal Gabor transformed images for blood vessel segmentation. This work starts with a 2-D Gabor filter for enhancing the retinal images to obtain the Gabor feature images. Subsequently, the Gabor feature images are enhanced by combining various enhancement techniques such as GCADW, homomorphic filter, JEH, unsharp masking filter, adaptive unsharp masking filter, and PSO unsharp masking filter. Segmentation is executed by utilizing hysteresis thresholding. The proposed methodology covers several stages. The proposed approach is summarized in Figure 1 as blocks that elaborate on each step.

The entire proposed segmentation process is described in detail as follows.

### 3.1. Gabor Filter

**Figure 2:**
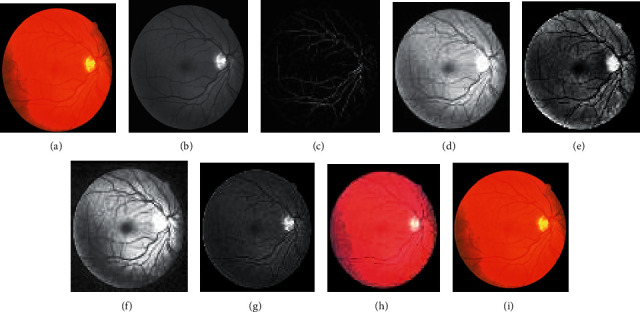
Images generated for retina 2 of the DRIVE data set by employing various enhancement techniques: (a) original, (b) green channel, (c) Gabor enhanced, (d) GCADW enhanced, (e) homomorphic filter enhanced, (f) JEH enhanced, (g) unsharp masking filter enhanced, (h) adaptive unsharp masking filter enhanced, and (i) PSO unsharp masking filter enhanced.

**Figure 3:**
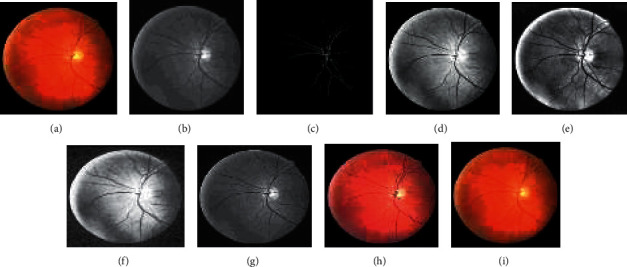
Images generated for retina 4 of the DRIVE data set by employing various enhancement techniques: (a) original, (b) green channel, (c) Gabor enhanced, (d) GCADW enhanced, (e) homomorphic filter enhanced, (f) JEH enhanced, (g) unsharp masking filter enhanced, (h) adaptive unsharp masking filter enhanced, and (i) PSO unsharp masking filter enhanced.

The approach activates by employing 2-D Gabor filters on the retinal images to achieve the Gabor transformed images. The Gabor transformed images are having object regions with enhanced boundaries. The Gabor kernel used in this work for producing *P*_*f*_(*x*, *y*) is defined as follows:

(23) $$\begin{array}{c} \psi_{u,v}\left(z\right)=\frac{\|k_{u,v}{\|}^{2}}{\sigma }\exp\left(-\frac{\|k_{u,v}\|z^{2}}{2\sigma^{2}}\right)\left[\exp\left(ik_{u,v}z\right)-\exp\left(\frac{-\sigma^{2}}{2}\right)\right], \end{array}$$

where the scale and orientation are determined by *u* and *v*, respectively. *z* = (*x*, *y*) .represents the norm operator. The wave vector *k*_*u*,*v*_=*k*_*v*_*e*^*jφu*^, where *k*_*v*_=*k*_max_/*λ*^*v*^ and *φ*_*u*_=*πu*/8. The parameter *λ* denotes the space among the filters in the frequency domain. The oscillatory part of the kernel is defined inside the first square bracket term, while the second term compensates the dc value of the kernel. The values of the Gabor kernel are selected as follows. *λ* = √2 for better intensification near the transition regions. The value of *k*_max_ = *π*/2, width = 60, height = 60, and *σ* = *π*/3. With parameters *k*_max_ and *λ*, Gabor filters of eight orientations are generated. Retinal Gabor transformed images are generated by convolving the retinal images with Gabor filters. Only the magnitude part is taken into consideration because the phase part is time varying in nature. Figures 2(a) and 2(b) and Figures 3(a) and 3(b) represent the original image and green channel image of retina 2, retina 4 of the DRIVE data set, and retina 5 of the CHASE_DB1 data set, respectively. Figures 2(c) and 3(c) illustrate the Gabor enhanced images of retina 2 and retina 4 of the DRIVE data set and retina 5 of the CHASE_DB1 data set, respectively. Afterward, hysteresis thresholding followed by morphological operation for cleaning is applied, and segmented images are obtained. All the parameter values are chosen on an experimental basis.

### 3.2. Various Enhancement Techniques

**Figure 4:**
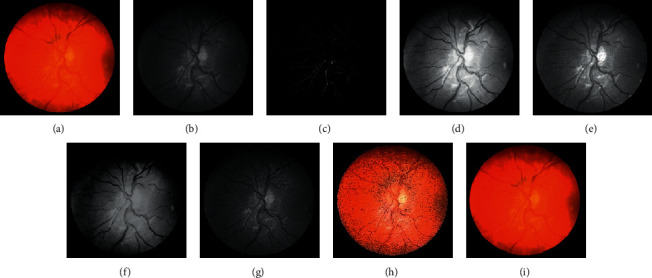
Images generated for retina 5 of the CHASE_DB1 data set by employing various enhancement techniques: (a) original, (b) green channel, (c) Gabor transformed, (d) GCADW enhanced, (e) homomorphic filter enhanced, (f) JEH enhanced, (g) unsharp masking filter enhanced, (h) adaptive unsharp masking filter enhanced, and (i) PSO unsharp masking filter enhanced.

**Figure 5:**
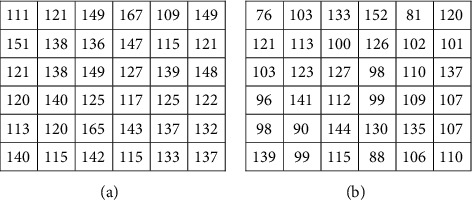
Description of joint equalization of histogram: (a) subimage representation and (b) average subimage representation.

In the subsequent step, investigations are carried out with the suggested approaches by integrating the enhanced methods with the Gabor filter. Generally, it is difficult to recommend one specific contrast-enhanced method that produces an output of free visual artifacts. Moreover, for verifying the quality of the output of the image, there are no particular reliable measures available in the literature. Therefore, choosing an appropriate algorithm to enhance the images is challenging. Accordingly, six enhancement methods are selected to improve the Gabor features. The algorithms of the suggested methods are explained below:

(i) GCADW enhancement is computed with three vital steps. The detailed mathematical computations of GCADW are described in (2)–(4) and (6). The steps of GCADW are summarized as follows:
  (a) Consider the image that is to be enhanced
  (b) Analyze the histogram of the image
  (c) In the next step, employ weighting distribution
  (d) Finally, apply gamma correction and obtain the enhanced image
Figures 2(d) and 3(d) represent the GCADW enhanced images of retina 2 and retina 4 of the DRIVE data set and retina 5 of the CHASE_DB1 data set, respectively.
(ii) The second enhancement technique is the homomorphic filter. The steps of the homomorphic filter are described below:
  (a) The multiplicative component described in (7) is converted to an additive component by applying the logarithm function
  (b) Apply Fourier transform on retinal images for converting the images into frequency-domain transformation
  (c) The transformed retinal images are processed through a homomorphic filter function as described in (8)
  (d) Take the inverse Fourier transform to obtain homomorphic filtered enhanced retinal images
The condition *λ*_*h*_ > *λ*_l_ > 0 must be followed while selecting the values of low- and high-frequency components. The soft edge and detail information may be eliminated if too small values are chosen for *λ*_*l*_. On the contrary, noise contained in high frequency may increase if large values of *λ*_*h*_ are chosen. In this work, the values are chosen as *h* = 0.8 and *λ*_*l*_ = 0.6, respectively. Figures 2(e), 3(e), and 4(e) represent the homomorphic filter enhanced images of retina 2 and retina 4 of the DRIVE data set and retina 5 of the CHASE_DB1 data set, respectively.
(iii) JEH enhanced technique deals with level pair of intensity and defined by the field of count as explained in (10), in which 256 × 256 is the order of the matrix. Utilizing (12), the equalized joint histogram is computed, and improved enhanced images are produced. The description of the JEH enhanced technique is as follows:
Figure 5(a) represents the intensities of a grayscale subimage *k* of 8 bit and size 6 × 6. By utilizing (11), the average subimage *M* is accomplished, and Figure 5(b) represents this. The size of the window is considered as three because the higher size window may blur the image, and according to the location, the pixel pairs are generated. For instance, both input and average images are represented in a pixel pair (1, 1) with values (111, 76). Among the pixel pairs, the minimum and maximum value specified by CDF is (109, 81) and (167, 152), respectively. The joint equalized histogram value is achieved by (12).
For example, the CDF of (140, 139) pixel pair is 11. The histogram equalized is calculated as follows:
  (24) $$\begin{array}{c} h_{eq}\left(140,139\right)=\text{round }\left(\frac{11-1}{35}\times 255\right)=\text{round}\left(0.285\times 255\right)=72. \end{array}$$
In the original subimage, the intensity value is substituted as 140, that is, *M* at every occurrence of the pixel pair (140, 139). In the rest of the original subimage spaces, the pixel pairs such as (140, 141) the value (140) is not substituted. In a similar manner, the rest of the equalized joint histogram values are computed. Figures 2(f) and 3(f) represent the JEH enhanced images of retina 2 and retina 4 of the DRIVE data set and retina 5 of the CHASE_DB1 data set, respectively.
(iv) The unsharp mask enhancement filter regulates the edge contrast and produces the illusion of a very intensive image. Thus, unsharp masking results in edge image *g*(*m*, *n*) transformed as a derivative of an input image *f*(*m*, *n*) as given below:
  (25) $$\begin{array}{c} g\left(m,n\right)=f\left(m,n\right)-f_{\text{smooth}}\left(m,n\right), \end{array}$$
where *f*_smooth_ (*m*, *n*) is a smooth version of *f*(*m*, *n*).
The final sharpening image obtained through unsharp masking is given as follows:
  (26) $$\begin{array}{c} f_{\text{sharp}}\left(m,n\right)=f\left(m,n\right)+k*g\left(m,n\right), \end{array}$$
where *k* is a scaling constant. Values of *k* vary between 0.2 and 0.7, with the higher values providing growing amounts of sharpening.
Figures 2(g) and 5(g) illustrate the unsharp masking filter enhanced images of retina 2 and retina 4 of the DRIVE data set and retina 5 of the CHASE_DB1 data set, respectively.
(v) The adaptive unsharp masking enhancement approach enhances the quality of the image with regard to the information volume, sharpness, and colourfulness by using equations (17), (18), and (20) as described in Section 2.6. Figures 2(h), 3(h), and 4(h) represent the adaptive unsharp masking filter enhanced images of retina 2 and retina 4 of the DRIVE data set and retina 5 of the CHASE_DB1 data set, respectively.
(vi) The algorithm for designing PSO-based unsharp masking filter enhancement technique is given below:
  (a) RGB input image
  (b) RGB colour space is converted to HSV colour space
  (c) PSO iteration count is set as zero
  (d) Consider kernel element and gain as a particle and initialize the particle
  (e) Repeat
  (f) kernel is generated from each particle
  (g) Unsharp masking filter operation is carried
  (h) Compute entropy penalized by over-range ration
  (i) Update the global best solution and particle motion
  (j) Particle position is updated until maximum iteration is touched
  (k) Return optimum solution, that is, global best solution
  (l) Finally, the edge extraction kernel and the augmentation gain factor are tuned using the PSO optimizer to yield contrast-enhanced images with minimum over-range artifacts.

Figures 2(i) and 3(i) represent the PSO unsharp masking filter enhanced images of retina 2 and retina 4 of the DRIVE data set and retina 5 of the CHASE_DB1 data set, respectively.

Figures 4(a) and 4(b) represent the original image and green channel image of retina 2 and retina 4 of the DRIVE data set, and retina 5 of the CHASE_DB1 data set, respectively.  Figure 4(c) represents the Gabor enhanced images of retina 2 and retina 4 of the DRIVE data set and retina 5 of the CHASE_DB1 data set.  Figure 4(d) illustrates the GCADW enhanced images of retina 2 and retina 4 of the DRIVE data set and retina 5 of the CHASE_DB1 data set.  Figure 4(f) represents the JEH enhanced images of retina 2 and retina 4 of the DRIVE data set and retina 5 of the CHASE_DB1 data set.  Figure 4(g) illustrates the unsharp masking filter enhanced images of retina 2 and retina 4 of the DRIVE data set and retina 5 of the CHASE_DB1 data set.  Figure 4(i) shows the PSO unsharp masking filter enhanced images of retina 2 and retina 4 of the DRIVE data set and retina 5 of the CHASE_DB1 data set.

**Figure 6:**
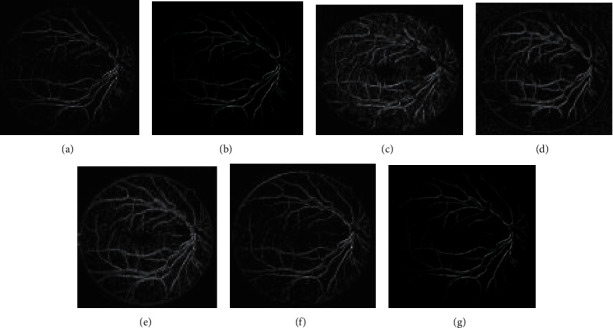
Images generated for retina 2 of the DRIVE data set by integrating the Gabor filter with different enhancement techniques: (a) original Gabor transformed image, (b) GCADW integrated with the Gabor filter, (c) homomorphic filter integrated with the Gabor filter, (d) JEH integrated with the Gabor filter, (e) unsharp masking filter integrated with the Gabor filter, (f) adaptive unsharp masking filter integrated with the Gabor filter, and (g) PSO unsharp masking filter integrated with the Gabor filter.

**Figure 7:**
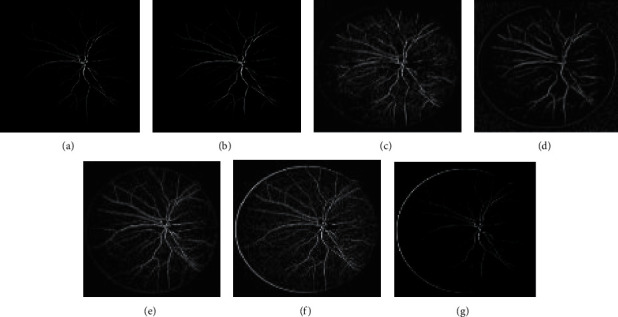
Images generated for retina 4 of the DRIVE data set by integrating Gabor filter with different enhancement techniques: (a) original Gabor transformed image, (b) GCADW integrated with the Gabor filter, (c) homomorphic filter integrated with the Gabor filter, (d) JEH integrated with the Gabor filter, (e) unsharp masking filter integrated with the Gabor filter, (f) adaptive unsharp masking filter integrated with the Gabor filter, and (g) PSO unsharp masking filter integrated with the Gabor filter.

**Figure 8:**
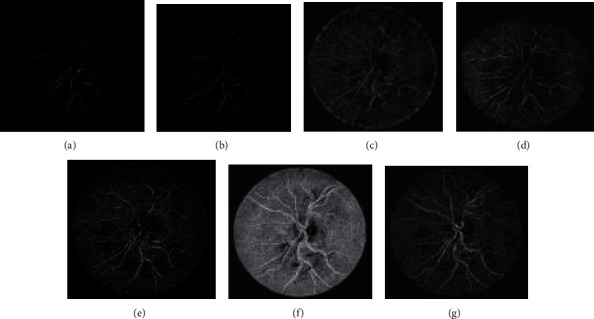
Images generated for retina 5 of the CHASE_DB1 data set by integrating Gabor filter with different enhancement techniques: (a) original Gabor transformed image, (b) GCADW integrated with the Gabor filter, (c) homomorphic filter integrated with the Gabor filter, (d) JEH integrated with the Gabor filter, (e) unsharp masking filter integrated with the Gabor filter, (f) adaptive unsharp masking filter integrated with the Gabor filter, and (g) PSO unsharp masking filter integrated with the Gabor filter.

Figures 6–8 display the images of the retina 2 and retina 4 of the DRIVE and retina 5 of the CHASE_DB1databases achieved from each enhancement technique integrated with the Gabor filter. From all the figures, it is distinctly noticeable that PSO unsharp masking filter integrated with the Gabor filter generates noise-free enhanced image in which the thick and thin vessels are visible.

## 4. Results and Discussion

The proposed idea is analyzed and examined on DRIVE (Digital Retinal Images for Vessel Extraction) and CHASE_DB1 (Child Heart and Health Study in England) databases. The DRIVE data set contains 20 coloured fundus images in each training and testing set, an equivalent set of masks, and two manually segmented sets. The first manual segmented image that first ophthalmologist provides is preserved as the ground truth image. In supervised models for training the network, the training data set is generally utilized. The test data set is utilized for the computation in this work as the proposed method is an unsupervised method. The ground truth images of the test data set are utilized for analysis purposes. The CHASE_DB1 data set consists of ground truth images of left and right eyes taken from 28 children. Comparisons between segmented and ground truth image are verified using metrics, that is, sensitivity (Sen), accuracy (Acc), and specificity (Sp).

(27) $$\begin{array}{c} \text{Sen}=\frac{\text{TP}}{\left(\text{TP}+\text{FN}\right)},\\ \text{Acc}=\frac{\left(\text{TP}+\text{TN}\right)}{\left(\text{TP}+\text{TN}+\text{FP}+\text{FN}\right)},\\ \text{Sp}=\frac{\text{TN}}{\left(\text{FP}+\text{TN}\right)}, \end{array}$$

where TP: true positive states as the correct identification of a vessel, TN: true negative states as the correct identification of a background, FP: false positive states as incorrect identification of a vessel, and FN: false negative states as incorrect identification of a background.

The measure of ability for verifying the correct vessel pixel is known as sensitivity. In contrast, the measure of the ability to verify accurate nonvessel pixels is known as specificity, and the accuracy displays the conventionality of the segmentation result.

Tables 2 and 3 present the performance of blood vessel segmentation using the original Gabor filter in terms of Sen, Acc, and Sp for DRIVE and CHASE_DB1. Traditional Gabor filter delivers Sen, Acc, and Sp as 0.6434, 0.9215, and 0.9470, respectively, on DRIVE and 0.6904, 0.9112, and 0.9349, respectively, on CHASE_DB1 databases. Tables 4 and 5 summarize the performance of GCADW integrated with the Gabor filter for blood vessel segmentation. The integrated proposed method attains Sen, Acc, and Sp as 0.6594, 0.9301, and 0.9502, respectively, on the DRIVE database and 0.7041, 0.9200, and 0.9375, respectively, on the CHASE_DB1 database. Tables 6 and 7 summarize the performance of the homomorphic filter combined with the Gabor filter for blood vessel segmentation. The integrated proposed method attains Sen, Acc, and Sp as 0.6377, 0.9292, and 0.9466, respectively, on the DRIVE database and 0.6489, 0.9174, and 0.9385, respectively, on the CHASE_DB1 database. Tables 8 and 9 summarize the performance of JHE integrated with the Gabor filter for blood vessel segmentation. The integrated suggested approach accomplishes Sen, Acc, and Sp as 0.6846, 0.9505, and 0.9620, respectively, on the DRIVE database and 0.7365, 0.9411, and 0.9501, respectively, on the CHASE_DB1 database. Tables 10 and 11 summarize the performance of the unsharp masking filter integrated with the Gabor filter for blood vessel segmentation. The integrated recommended technique accomplishes Sen, Acc, and Sp as 0.6408, 0.9512, and 0.9528, respectively, on the DRIVE database and 0.6757, 0.9132, and 0.9334, respectively, on the CHASE_DB1 database. Tables 12 and 13 summarize the performance of the adaptive unsharp masking filter integrated with the Gabor filter for blood vessel segmentation. The integrated proposed method accomplishes Sen, Acc, and Sp as 0.7207, 0.9518, and 0.9604, respectively, on the DRIVE database and 0.7357, 0.9535, and 0.9741, respectively, on the CHASE_DB1 database. Tables 14 and 15 summarize the performance of the PSO unsharp masking filter integrated with the Gabor filter for blood vessel segmentation. The integrated proposed method accomplishes Sen, Acc, and Sp as 0.7482, 0.95934, and 0.9801, respectively, on the DRIVE database and 0.7594, 0.9612, and 0.9842, respectively, on the CHASE_DB1 database.

In view of the DRIVE database, the suggested approaches report a substantial improvement in Sp and Acc scores. However, the suggested approaches report moderate Sen scores. Considering the CHASE_DB1 database, the suggested approaches report a substantial improvement in Sen, Acc, and Sp scores. It is also noteworthy here that in terms of accuracy, the suggested approaches register a substantial improvement for both the databases.

The entire segmentation approach can be categorized into under-segmentation, over-segmentation, or accurate segmentation.

- If specificity shows a higher side and sensitivity shows a lower side, then the vessel is undersegmented that may result in the vessel being inaccurately recognized
- If specificity shows the lower side and sensitivity shows the higher side, then the vessel is oversegmented and may lead to nonvessel being recognized as a vessel
- If sensitivity and specificity are higher, then the vessel is segmented accurately

From all the tables, it is inferred that:

- For the DRIVE data set, the proposed homomorphic filter integrated with the Gabor filter technique underperforms the traditional Gabor filter in terms of all the performance measures. Similarly, the proposed sharpen filter integrated with the Gabor filter technique underperforms of sensitivity than the original Gabor filter. However, the rest all the recommended approaches outperform the original Gabor filter. The highest performance measures are achieved by PSO sharpen filter integrated with the Gabor filter.
- For the CHASE_DB1 data set, two suggested methods homomorphic filter integrated with the Gabor filter and unsharp masking filter combined with the Gabor filter underperform the traditional Gabor filter in terms of all the performance measures. However, remaining all the recommended approaches outperform the original Gabor filter. Like the DRIVE data set, the highest performance measures are achieved by the PSO unsharp masking filter integrated with the Gabor filter.

**Figure 9:**
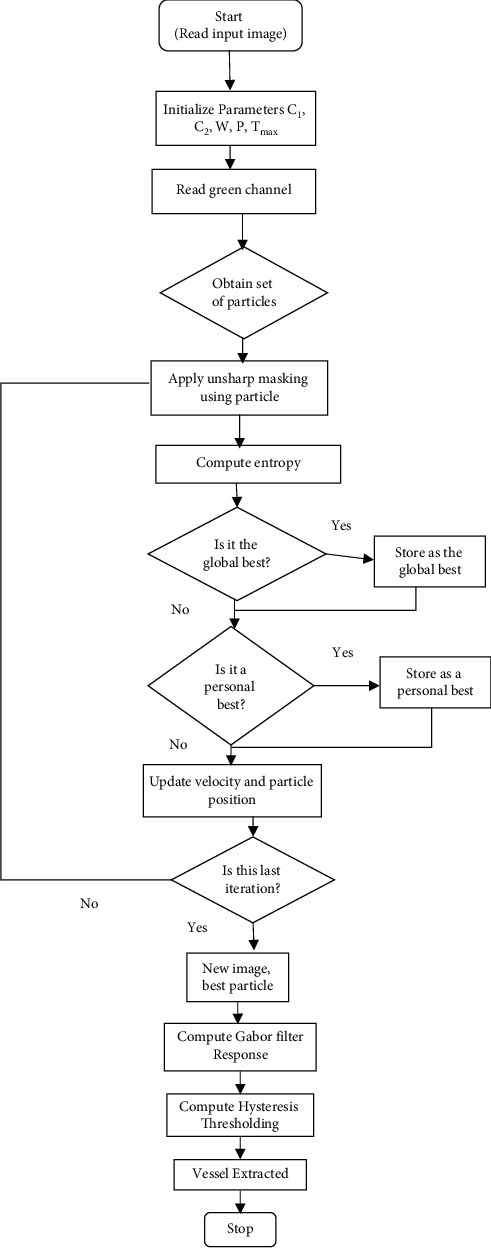
Flowchart of the final algorithm.

Overall, it can be deduced from the above discussion that all the enhancement variations that are adopted for Gabor filter yield better performance measures either with regard to Acc or Sen or Sp.  Figure 9 illustrates the flowchart of the final algorithm of the suggested approach.

**Figure 10:**
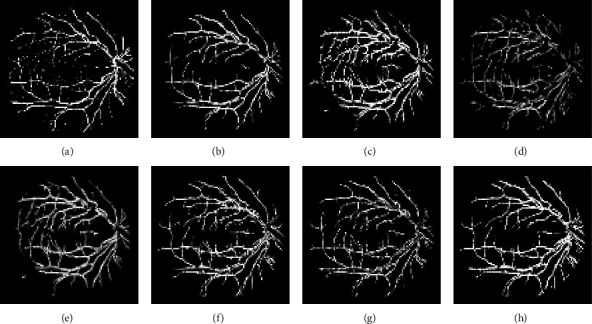
Segmented images achieved for various integrated techniques for retina 2 of the DRIVE data set: (a) ground truth image, (b) original Gabor transformed image, (c) Gabor integrated with GCADW, (d) Gabor integrated with homomorphic filter, (e) Gabor integrated with JHE, (f) Gabor integrated with sharpen filter, (g) Gabor integrated with adaptive sharpen filter, and (h) Gabor integrated with PSO sharpen filter.

**Figure 11:**
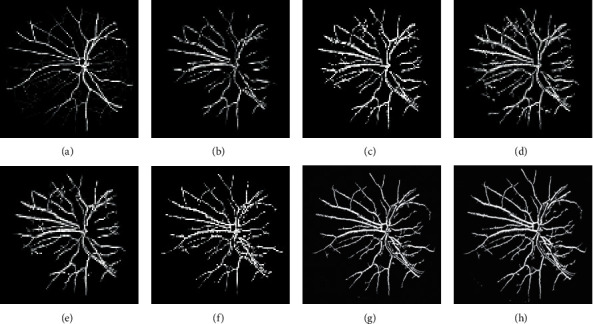
Segmented images achieved for various integrated techniques for retina 4 of the DRIVE data set: (a) ground truth image, (b) original Gabor transformed image, (c) Gabor integrated with GCADW, (d) Gabor integrated with homomorphic filter, (e) Gabor integrated with JHE, (f) Gabor integrated with sharpen filter, (g) Gabor integrated with adaptive sharpen filter, and (h) Gabor integrated with PSO sharpen filter.

**Figure 12:**
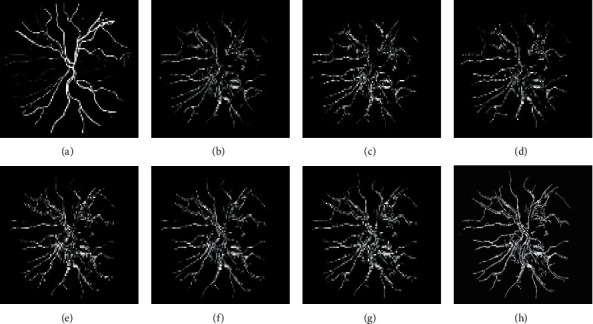
Segmented images achieved for various integrated techniques for retina 5 of the CHASE_DB1 data set: (a) Ground truth image, (b) original Gabor transformed image, (c) Gabor integrated with GCADW, (d) Gabor integrated with homomorphic filter, (e) Gabor integrated with JHE, (f) Gabor integrated with sharpen filter, (g) Gabor integrated with adaptive sharpen filter, and (h) Gabor integrated with PSO sharpen filter.

The segmented images of the retina 2 and retina 4 of the DRIVE database and retina 5 of the CHASE_DB1database achieved from different suggested approaches are represented in Figures 10–12, respectively.

The explanations of both the algorithms are as follows. First, read the colour image and extract the green channel of the image. Next, initialize all the parameters of PSO-based unsharp masking filter. Utilizing the global best solution, the unsharp mask image is generated. In the next step, the Gabor filter is applied, and a maximum Gabor enhanced image is generated. In the last step, hysteresis thresholding is applied with morphological cleaning for the vessel extraction.

To better explain the proposed idea's superiority, we have compared it with various state-of-the-art methods from the literature and given the results of experiments in Table 16. It represents the comparison with different suggested approaches by using mentioned metrics: sensitivity, accuracy, and specificity for models presented in Cinsdikici and Aydın [26], Zhang et al. [27], Rawi et al. [29], Rawi and Karajeh [30], Sreejini and Govindan [31], Chaudhari et al. [32], Soares et al. [33], Shabbir et al. [34], Aguirre-Ramos et al. [35], Yavuz and Kose [36], Farokhian et al. [37], Sundaram et al. [38], Dash et al. [36], Primitivo et al. [41], Hashemzadeh and Azar [42], Khawaja et al. [44], and Wang et al. [45]. The results of all the proposed models are summarized in Table 15. After a comprehensive study of Table 16, it concludes that among all the suggested approaches, the PSO unsharp masking filter integrated with the Gabor filter delivers the highest accuracy, that is, 0.959 for the DRIVE data set and 0.961 for the CHASE_DB1 data set. Furthermore, it is observed that the suggested method delivers better results than many state-of –art-of-methods and outperforms the existing Gabor filter technique.

## 5. Conclusions

In this work, six enhancement techniques are individually combined with the Gabor filter to improve the performance of the standard Gabor filter. The proposed techniques are assessed using DRIVE and CHASE_DB1 data sets. All together six algorithms are recommended for the improvement of the traditional Gabor filter. The parameters Sen, Acc, and Sp are taken into account in order to determine the best algorithm. Experimental results are compared with state-of-the-art models. It is observed that the homomorphic filter and unsharp masking filter integrated with the Gabor filter underperforms compared to the standard Gabor filter in terms of sensitivity on the DRIVE database. Similarly, homomorphic and unsharp masking filters combined with the Gabor filter underperform the standard Gabor filter in all performance measures on the CHASE_DB1 database. The best results are attained with a PSO unsharp masking filter with the Gabor filter by delivering an average value of Sen, Acc, and Sp of 0.748, 0.959, and 0.9801 on the DRIVE data set, respectively, and 0.759, 0.961, and 0.984 on the CHASE_DB1 data set, respectively. Therefore, it is inferred that adding different enhancement techniques before Gabor filter boosts the performance of the traditional Gabor filter and also improves the accuracy, specifically with respect to the tiny vessels.

Consequently, it is observed that though deep learning, a supervised approach is actively implemented for blood vessel extraction in recent research and achieving better results; still, the unsupervised traditional methods can be enhanced to achieve precise vessel segmentation. Also, the results of the suggested approach that is an unsupervised approach outperform many state-of-the-art methods that are coming under the group of unsupervised approaches.

Moreover, it will enable new practical applications, where analysis of low-contrast images in real time is required, for example, robotic microsurgery of the eye.

A drawback of the suggested model is that even though six enhancement techniques are combined with the Gabor filter, but only one integrated model is able to perform better as compared to the other integrated models. This is because enhancement of certain features might be accompanied by undesirable effects that might be led to the loss of valuable image information.

For future studies, we suggest considering different illumination normalization techniques such as small-scale retinex (SSR), multi-scale retinex (MSR), isotropic illumination, wavelet normalization, and so on combined with deep learning approaches for vessel segmentation.

## Figures and Tables

**Table 1:** Summary of advantages and disadvantages of vessel segmentation methods.

Methods and References	Data set	Advantages	Disadvantages
Extended median filter [23]	DRIVE	Simple to implement	Less accuracy
Blood vessels segmentation based on simplified PCNN and fast 2D-otsu algorithm [24]	DRIVE	Performed better in small vessels with high accuracy	Cannot remove pathological regions with low specificity
Clifford matched filter [25]	DRIVE	High accuracy	Low sensitivity
Using MF/ant (matched filter/ant colony) [26]	DRIVE	High accuracy	Cannot remove pathological regions with low specificity
Improved matched filter [29]	DRIVE	High accuracy	Low specificity
Genetic algorithm matched filter optimization [30]	DRIVE	High sensitivity	Low accuracy
Improved multi-scale matched filter using PSO algorithm [31]	DRIVE	High sensitivity	Low specificity and accuracy
Segmentation of retinal vessels by the use of Gabor wavelet and linear mean-squared-error classifier [32]	DRIVE	—	Very low accuracy
Gabor filters with the imperialism competitive algorithm [37]	DRIVE	High sensitivity and specificity	Low accuracy
Hybrid segmentation approach [38]	DRIVE	High accuracy	Very low sensitivity
A hybrid method to enhance thick and thin vessels [39]	DRIVE	High sensitivity and specificity	Low accuracy
A hybrid method for blood vessel segmentation [41]	DRIVE	—	Very high accuracy, sensitivity, and specificity
Frangi filter coupled with the probabilistic patch-based denoiser [44]	DRIVE	High sensitivity	Low accuracy
A context spatial U-Net for accurate blood vessel segmentation [45]	DRIVE	High sensitivity and specificity	Low accuracy

**Table 2:** Performance matrices results of original Gabor filter on the DRIVE database.

Fundus images	Sen	Acc	Sp
FI01	0.677887	0.918439	0.942004
FI02	0.664339	0.925255	0.955022
FI03	0.672271	0.904431	0.930137
FI04	0.570534	0.934659	0.971549
FI05	0.639169	0.927534	0.957341
FI06	0.656184	0.921006	0.949561
FI07	0.657170	0.928000	0.955230
FI08	0.666138	0.914571	0.937958
FI09	0.686661	0.916338	0.936594
FI10	0.634372	0.932265	0.95898
FI11	0.634372	0.922985	0.953728
FI12	0.634372	0.918548	0.939569
FI13	0.634372	0.922872	0.956796
FI14	0.634372	0.912605	0.928476
FI15	0.634372	0.931861	0.951623
FI16	0.634372	0.918399	0.946777
FI17	0.634372	0.917223	0.943944
FI18	0.634372	0.915814	0.938357
FI19	0.634372	0.922488	0.944618
FI20	0.634372	0.925097	0.943015
Average value	0.643422	0.921519	0.947063

Note: FI represents the number of fundus images of the corresponding database.

**Table 3:** Performance matrices results of original Gabor filter on the CHASE_DB1 database.

Fundus images	Sen	Acc	Sp
FI01	0.710047	0.904558	0.930882
FI02	0.682305	0.902526	0.926700
FI03	0.667443	0.916788	0.950170
FI04	0.699412	0.918843	0.941316
FI05	0.692741	0.901190	0.932334
FI06	0.682634	0.917574	0.939770
FI07	0.695286	0.904910	0.930149
FI08	0.695286	0.904910	0.930149
FI09	0.678926	0.921164	0.950061
FI10	0.691368	0.919804	0.937123
FI11	0.690519	0.904957	0.936737
FI12	0.691258	0.919311	0.917941
FI13	0.696819	0.910267	0.933915
FI14	0.692166	0.910005	0.932175
Average value	0.690443	0.911200	0.934958

**Table 4:** Performance matrices results of GCADW integrated with the Gabor filter on the DRIVE database.

Fundus images	Sen	Acc	Sp
FI01	0.679450	0.938196	0.947585
FI02	0.674608	0.925476	0.954097
FI03	0.678351	0.929122	0.954675
FI04	0.673793	0.935613	0.970244
FI05	0.684191	0.929685	0.961264
FI06	0.643044	0.923909	0.954194
FI07	0.644136	0.938770	0.957396
FI08	0.658671	0.932624	0.947472
FI09	0.677910	0.923463	0.945119
FI10	0.630137	0.930834	0.957801
FI11	0.631023	0.924642	0.955479
FI12	0.676946	0.934908	0.945506
FI13	0.612728	0.921469	0.937949
FI14	0.684116	0.912605	0.928476
FI15	0.667044	0.930416	0.948405
FI16	0.620389	0.921463	0.951344
FI17	0.626346	0.937751	0.944616
FI18	0.664990	0.932448	0.943742
FI19	0.664718	0.930584	0.953729
FI20	0.696847	0.948388	0.945688
Average value	0.659471	0.930118	0.950239

**Table 5:** Performance matrices results of GCADW integrated with the Gabor filter on the CHASE_DB1 database.

Fundus images	Sen	Acc	Sp
FI01	0.715652	0.921977	0.945813
FI02	0.704742	0.917989	0.929782
FI03	0.695609	0.920585	0.926741
FI04	0.699744	0.890291	0.939458
FI05	0.693052	0.928501	0.938527
FI06	0.705556	0.915255	0.935383
FI07	0.708359	0.924627	0.934857
FI08	0.693451	0.911141	0.934172
FI09	0.696969	0.926043	0.953173
FI10	0.707692	0.924176	0.948024
FI11	0.708923	0.919948	0.941466
FI12	0.704802	0.923779	0.940930
FI13	0.713273	0.928199	0.934405
FI14	0.710256	0.927990	0.923165
Average value	0.704148	0.920035	0.937564

**Table 6:** Performance matrices results of homomorphic filter integrated with the Gabor filter on the DRIVE database.

Fundus images	Sen	Acc	Sp
FI01	0.642459	0.910447	0.944741
FI02	0.634353	0.931709	0.944494
FI03	0.628193	0.924788	0.959878
FI04	0.626216	0.928484	0.952147
FI05	0.638631	0.937319	0.958194
FI06	0.649660	0.944162	0.952682
FI07	0.634286	0.939462	0.944454
FI08	0.641661	0.932704	0.947472
FI09	0.631430	0.925241	0.940213
FI10	0.634540	0.923662	0.944142
FI11	0.636221	0.946333	0.947808
FI12	0.631881	0.929728	0.950315
FI13	0.631964	0.926854	0.942059
FI14	0.648458	0.921683	0.948679
FI15	0.638458	0.921683	0.958679
FI16	0.639647	0.935233	0.933576
FI17	0.64563	0.911425	0.936851
FI18	0.631460	0.937980	0.940054
FI19	0.645855	0.921394	0.943233
FI20	0.644290	0.934790	0.943087
Average value	0.637764	0.929254	0.946630

**Table 7:** Performance matrices results of homomorphic integrated with the Gabor filter on the CHASE_DB1 database.

Retinal images	Sen	Acc	Sp
FI01	0.644795	0.949065	0.936870
FI02	0.643583	0.928927	0.933554
FI03	0.636338	0.926789	0.936815
FI04	0.639947	0.917553	0.943018
FI05	0.648564	0.913205	0.947289
FI06	0.645292	0.913339	0.937429
FI07	0.655333	0.919822	0.934125
FI08	0.653901	0.905724	0.930070
FI09	0.650733	0.919650	0.952101
FI10	0.657497	0.905404	0.938351
FI11	0.648734	0.904013	0.938093
FI12	0.649968	0.914724	0.944650
FI13	0.640965	0.911713	0.937150
FI14	0.669859	0.914765	0.930609
Average value	0.648964	0.917478	0.93858

**Table 8:** Performance matrices results of JEH integrated with the Gabor filter on the DRIVE database.

Fundus images	Sen	Acc	Sp
FI01	0.667269	0.948305	0.955836
FI02	0.679432	0.945257	0.961289
FI03	0.692271	0.954431	0.950137
FI04	0.680534	0.944659	0.971549
FI05	0.699169	0.937534	0.957341
FI06	0.686180	0.951006	0.969561
FI07	0.697170	0.948547	0.955238
FI08	0.696138	0.944571	0.957958
FI09	0.696661	0.956338	0.966594
FI10	0.634372	0.952265	0.95898
FI11	0.680312	0.962985	0.953728
FI12	0.696104	0.958548	0.959569
FI13	0.669814	0.952872	0.956796
FI14	0.732166	0.962605	0.968476
FI15	0.675489	0.951861	0.961623
FI16	0.682473	0.958399	0.976777
FI17	0.677388	0.947223	0.963944
FI18	0.67384	0.945814	0.968357
FI19	0.677834	0.942488	0.964618
FI20	0.699361	0.945097	0.963015
Average value	0.684698	0.950540	0.962060

**Table 9:** Performance matrices results of JEH integrated with the Gabor filter on the CHASE_DB1 database.

Fundus images	Sen	Acc	Sp
FI01	0.745752	0.945233	0.950308
FI02	0.712943	0.943283	0.941410
FI03	0.722359	0.938871	0.944343
FI04	0.734211	0.941279	0.945253
FI05	0.730888	0.930656	0.947073
FI06	0.744306	0.938812	0.953431
FI07	0.739790	0.944063	0.949192
FI08	0.747950	0.938944	0.949204
FI09	0.734789	0.946909	0.954734
FI10	0.748618	0.935006	0.944861
FI11	0.731055	0.946087	0.959027
FI12	0.746762	0.946433	0.956740
FI13	0.735172	0.944196	0.957555
FI14	0.736517	0.936948	0.949115
Average value	0.736508	0.941194	0.950160

**Table 10:** Performance matrices results of unsharp masking filter integrated with the Gabor filter on the DRIVE database.

Retinal images	Sen	Acc	Sp
FI01	0.642745	0.959207	0.956494
FI02	0.634238	0.958333	0.950477
FI03	0.642644	0.949351	0.954470
FI04	0.638033	0.958249	0.953599
FI05	0.632061	0.958413	0.952844
FI06	0.649474	0.951926	0.956991
FI07	0.630450	0.942967	0.953198
FI08	0.637316	0.948850	0.957821
FI09	0.648731	0.953721	0.950917
FI10	0.639051	0.941742	0.940817
FI11	0.640097	0.958853	0.962328
FI12	0.632187	0.946134	0.952573
FI13	0.659457	0.958680	0.934602
FI14	0.645615	0.951776	0.956392
FI15	0.636682	0.959714	0.95736
FI16	0.644005	0.955084	0.956033
FI17	0.637276	0.949379	0.954395
FI18	0.648286	0.942240	0.959790
FI19	0.633002	0.925624	0.948454
FI20	0.645038	0.955596	0.948340
Average value	0.640819	0.951291	0.952894

**Table 11:** Performance matrices results of unsharp masking filter integrated with the Gabor filter on the CHASE_DB1 database.

Fundus images	Sen	Acc	Sp
FI01	0.641601	0.914316	0.933804
FI02	0.670786	0.924334	0.932685
FI03	0.659763	0.920059	0.931729
FI04	0.672605	0.914752	0.936606
FI05	0.672497	0.915543	0.939502
FI06	0.675362	0.911253	0.935985
FI07	0.669027	0.902999	0.922259
FI08	0.698382	0.908980	0.934595
FI09	0.686771	0.912538	0.931902
FI10	0.698150	0.914770	0.936312
FI11	0.680958	0.915039	0.936026
FI12	0.698290	0.903039	0.931026
FI13	0.669055	0.913874	0.932498
FI14	0.667872	0.914562	0.933582
Average value	0.675794	0.913289	0.933460

**Table 12:** Performance matrices results of adaptive unsharp masking filter integrated with the Gabor filter on the DRIVE database.

Fundus images	Sen	Acc	Sp
FI01	0.728404	0.952835	0.958943
FI02	0.724877	0.947196	0.962225
FI03	0.702186	0.953249	0.964470
FI04	0.716533	0.950525	0.968415
FI05	0.711223	0.95876	0.958583
FI06	0.719649	0.950552	0.955150
FI07	0.717170	0.947410	0.965238
FI08	0.736097	0.954036	0.958023
FI09	0.718266	0.953270	0.960487
FI10	0.728316	0.950265	0.956447
FI11	0.730081	0.953082	0.956807
FI12	0.723043	0.956661	0.95819
FI13	0.734215	0.950947	0.968519
FI14	0.707463	0.959728	0.964880
FI15	0.729155	0.940718	0.962421
FI16	0.710953	0.955808	0.967056
FI17	0.720151	0.950332	0.952475
FI18	0.713341	0.952477	0.962521
FI19	0.715501	0.951121	0.954244
FI20	0.728393	0.948643	0.954857
Average value	0.720750	0.951880	0.960497

**Table 13:** Performance matrices results of adaptive unsharp masking filter integrated with the Gabor filter on the CHASE_DB1 database.

Fundus images	Sen	Acc	Sp
FI01	0.716373	0.955950	0.993391
FI02	0.728609	0.952957	0.991640
FI03	0.731022	0.958210	0.994449
FI04	0.733234	0.958449	0.995169
FI05	0.737886	0.958862	0.961610
FI06	0.749893	0.952881	0.982098
FI07	0.734097	0.950622	0.983816
FI08	0.734952	0.954432	0.948331
FI09	0.735258	0.950659	0.931450
FI10	0.747998	0.946681	0.985081
FI11	0.736358	0.951839	0.924037
FI12	0.735460	0.957028	0.985096
FI13	0.747984	0.945325	0.981843
FI14	0.730824	0.955117	0.979578
Average value	0.735710	0.953500	0.974110

**Table 14:** Performance matrices results of PSO unsharp masking filter integrated with the Gabor filter on the DRIVE database.

Fundus images	Sen	Acc	Sp
FI01	0.746162	0.965569	0.979961
FI02	0.748538	0.958730	0.977710
FI03	0.730468	0.958861	0.975244
FI04	0.748574	0.955770	0.987287
FI05	0.748794	0.959573	0.980304
FI06	0.749458	0.954015	0.980577
FI07	0.734503	0.956558	0.979877
FI08	0.749806	0.959388	0.968673
FI09	0.756975	0.962064	0.988955
FI10	0.74559	0.963892	0.978787
FI11	0.741653	0.957203	0.979945
FI12	0.749106	0.957582	0.982605
FI13	0.749206	0.959318	0.977894
FI14	0.745921	0.956851	0.987419
FI15	0.756153	0.958874	0.968376
FI16	0.758354	0.958772	0.980668
FI17	0.748113	0.958524	0.978776
FI18	0.750263	0.958339	0.981894
FI19	0.753979	0.967158	0.987246
FI20	0.753445	0.959777	0.980127
Average value	0.748200	0.959340	0.980110

**Table 15:** Performance matrices results of PSO unsharp masking filter integrated with the Gabor filter on the CHASE_DB1 database.

Fundus images	Sen	Acc	Sp
FI01	0.759308	0.960156	0.994205
FI02	0.752015	0.954933	0.994897
FI03	0.765499	0.962807	0.985968
FI04	0.765182	0.965952	0.991941
FI05	0.757867	0.967832	0.978686
FI06	0.765903	0.956606	0.988225
FI07	0.752763	0.958306	0.981853
FI08	0.762151	0.963436	0.975938
FI09	0.750780	0.958976	0.977082
FI10	0.759728	0.968681	0.977371
FI11	0.756047	0.969477	0.979326
FI12	0.759391	0.954646	0.987873
FI13	0.759015	0.956353	0.978796
FI14	0.766664	0.95988	0.986658
Average value	0.759450	0.961280	0.984201

**Table 16:** The average results from DRIVE and CHASE_DB1 data sets were compared with some other approaches.

Approaches	DRIVE		CHASE_DB1			
	Sen	Acc	Sp	Sen	Acc	Sp
Cinsdikici and Aydın [26]	—	0.929	—	—	—	—
Zhang et al. [27]	0.712	0.938	—	—	—	—
Rawi et al. [29]	—	0.953	—	—	—	—
Rawi and Karajeh [30]	—	0.942	—	—	—	—
Sreejini and Govindan [31]	0.713	0.963	0.986	—	—	—
Chaudhari et al. [32]		0.867	—	—	—	—
Soares et al. [33]	—	0.946	—	—	—	—
Shabbir et al. [34]	—	0.950	—	—	—	—
Aguirre-Ramos et al. [35]	0.785	0.950	0.966	—	—	—
Yavuz and Kose [36]	0.677	0.957	0.978	—	—	—
Farokhian et al. [37]	0.693	0.939	0.979	—	—	—
Sundaram et al. [38]	0.690	0.930	0.940	0.710	0.950	0.960
Dash et al. [36]	0.756	0.952	0.981	0.770	0.950	0.970
Primitivo et al. [41]	0.846	0.961	0.970	—	—	—
Hashemzadeh and Azar [42]	0.783	0.953	0.980	0.773	0.962	0.984
Khawaja et al. [44]	0.802	0.956	0.973	—	—	—
Wang et al. [45]	**0.807**	0.956	0.978	**0.842**	0.970	0.982
Original Gabor filter	0.643	0.921	0.947	0.690	0.911	0.934
Proposed GCADW integrated with the Gabor filter	0.659	0.930	0.950	0.704	0.920	0.937
Proposed homomorphic filter integrated with the Gabor filter	0.637	0.929	0.946	0.648	0.917	0.938
Proposed JHE integrated with the Gabor filter	0.684	0.950	0.962	0.736	0.941	0.950
Proposed unsharp masking filter integrated with the Gabor filter	0.640	0.951	0.952	0.675	0.913	0.933
Proposed adaptive unsharp masking filter integrated with the Gabor filter	0.720	0.951	0.960	0.735	0.953	0.974
Proposed PSO unsharp masking filter integrated with the Gabor filter	0.748	**0.959**	**0.980**	0.759	**0.961**	**0.984**

## Data Availability

Publicly available data are used in this study.

## References

[1] Abramoff M. D., Garvin M. K., Sonka M. (2010). Retinal imaging and image analysis. *IEEE Reviews in Biomedical Engineering*.

[2] Liew G., Wang J. J. (2011). Retinal vascular signs: a window to the heart?. *Revista Española de Cardiología*.

[3] Narasimhan K., Neha V. C., Vijayarekha K. (2012). Hypertensive retinopathy diagnosis from fundus images by estimation of Avr. *Procedia Engineering*.

[4] Al-Diri B., Hunter A., Steel D. (2009). An active contour model for segmenting and measuring retinal vessels. *IEEE Transactions on Medical Imaging*.

[5] Chaudhuri S., Chatterjee S., Katz N., Nelson M., Goldbaum M. (1989). Detection of blood vessels in retinal images using two-dimensional matched filters. *IEEE Transactions on Medical Imaging*.

[6] Roy S., Whitehead T. D., Li S. (Jan 2022). Co-clinical FDG-PET radiomic signature in predicting response to neoadjuvant chemotherapy in triple-negative breast cancer. *European Journal of Nuclear Medicine and Molecular Imaging*.

[7] Hoover A. D., Kouznetsova V., Goldbaum M. (2000). Locating blood vessels in retinal images by piecewise threshold probing of a matched filter response. *IEEE Transactions on Medical Imaging*.

[8] Staal J., Abramoff M. D., Niemeijer M., Viergever M. A., Van Ginneken B. (2004). Ridge-based vessel segmentation in color images of the retina. *IEEE Transactions on Medical Imaging*.

[9] Roy S., Shoghi K. I., Karray F., Campilho A., Yu A. (2019). Computer-aided tumor segmentation from T2-weighted MR images of patients derived tumor xenografts. *Image Analysis and Recognition. ICAR 2019*.

[10] Xiaoyi Jiang X., Mojon D. (2003). Adaptive local thresholding by verification-based multithreshold probing with application to vessel detection in retinal images. *IEEE Transactions on Pattern Analysis and Machine Intelligence*.

[11] Azzopardi G., Strisciuglio N., Vento M., Petkov N. (2015). Trainable COSFIRE filters for vessel delineation with application to retinal images. *Medical Image Analysis*.

[12] Mapayi T., Viriri S., Tapamo J.-R. (Feb 2015). Comparative study of retinal vessel segmentation based on global thresholding techniques. *Computational and Mathematical Methods in Medicine*.

[13] Strisciuglio N., Azzopardi G., Vento M., Petkov N. Multiscale blood vessel delineation using B-COSFIRE filters.

[14] Roy S., Whitehead T. D., Quirk J. D. (Sep 2020). Optimal co-clinical radiomics: sensitivity of radiomic features to tumour volume, image noise and resolution in co-clinical T1-weighted and T2-weighted magnetic resonance imaging. *EBioMedicine*.

[15] Rajput Y., manza R., Patwari M., Deshpande N., Jalgaon M. “Retinal blood vessels extraction using 2D median filter.

[16] Maison, Lestari T., Luthfi A. (2019). Retinal blood vessel segmentation using Gaussian filter. *Journal of Physics: Conference Series*.

[17] Malarvel M., Nayak S. R. (1 Jan.2020). Edge and region segmentation in high-resolution aerial images using improved kernel density estimation: a hybrid approach. *Journal of Intelligent and Fuzzy Systems*.

[18] Kaur J., Sinha H. P. (2012). Automated detection of retinal blood vessels in diabetic retinopathy using Gabor filter. *Int. J of Comp Sc and Net.*.

[19] Bao X.-R., Ge X., She L.-H., Zhang S. (2015). Segmentation of retinal blood vessels based on cake filter. *BioMed Research International*.

[20] Chatterjee S., Dutta R. K., Ganguly D., Chatterjee K., Roy S., Tiwary U., Chaudhury S. (2019). “Bengali Handwritten Character Classification Using Transfer Learning on Deep Convolutional Network. *Intelligent Human Computer Interaction. IHCI 2019*.

[21] Kochner B., Schuhmann D., Michaelis M., Mann G., Englmeier K.-H. Course tracking and contour extraction of retinal vessels from color fundus photographs: most efficient use of steerable filters for model-based image analysis.

[22] Sabaz F., Atila U. (2017). ROI detection and vessel segmentation in retinal image. *The International Archives of the Photogrammetry, Remote Sensing and Spatial Information Sciences*.

[23] Dash S., Sahu G. Retinal blood vessel segmentation by employing various upgraded median filters.

[24] Yao C., Chen H.-j. (2009). Automated retinal blood vessels segmentation based on simplified PCNN and fast 2D-otsu algorithm. *Journal of Central South University of Technology*.

[25] Roy S., Mitra A., Roy S., Setua S. K. (Dec 2019). Blood vessel segmentation of retinal image using Clifford matched filter and Clifford convolution. *Multimedia Tools and Applications*.

[26] Cinsdikici M. G., Aydın D. (2009). Detection of blood vessels in ophthalmoscope images using MF/ant (matched filter/ant colony) algorithm. *Computer Methods and Programs in Biomedicine*.

[27] Zhang B., Zhang L., Zhang L., Karray F. (2010). Retinal vessel extraction by matched filter with first-order derivative of Gaussian. *Computers in Biology and Medicine*.

[28] Roy S., Bhattacharyya D., Bandyopadhyay S. K., Kim T.-H. (Jun 2017). An iterative implementation of level set for precise segmentation of brain tissues and abnormality detection from MR images. *IETE Journal of Research*.

[29] Al-Rawi M., Qutaishat M., Arrar M. (2007). An improved matched filter for blood vessel detection of digital retinal images. *Computers in Biology and Medicine*.

[30] Al-Rawi M., Karajeh H. (2007). Genetic algorithm matched filter optimization for automated detection of blood vessels from digital retinal images. *Computer Methods and Programs in Biomedicine*.

[31] Sreejini K. S., Govindan V. K. (2015). Improved multiscale matched filter for retina vessel segmentation using PSO algorithm. *Egyptian Informatics Journal*.

[32] Chaudhari H. P., Rahulkar A. D., Patil C. Y. (2014). Segmentation of retinal vessels by the use of gabor wavelet and linear mean squared error classifier. *Int. .J Emerg. Res. Tech.*.

[33] Soares J. V. B., Leandro J. J. G., Cesar R. M., Jelinek H. F., Cree M. J. (2006). Retinal vessel segmentation using the 2-D Gabor wavelet and supervised classification. *IEEE Transactions on Medical Imaging*.

[34] Shabbir S., Tariq A., Akram M. U. (2013). A Comparison and evaluation of computerized methods for blood vessel enhancement and segmentation in retinal images. *International Journal of Future Computer and Communication*.

[35] Aguirre-Ramos H., Avina-Cervantes J. G., Cruz-Aceves I., Ruiz-Pinales J., Ledesma S. (2018). Blood vessel segmentation in retinal fundus images using Gabor filters, fractional derivatives, and Expectation Maximization. *Applied Mathematics and Computation*.

[36] Yavuz Z., Köse C. (2017). Blood vessel extraction in color retinal fundus images with enhancement filtering and unsupervised classification. *Journal of Healthcare Engineering*.

[37] Farokhian F., Yang C., Demirel H., Wu S., Beheshti I. (2017). Automatic parameters selection of Gabor filters with the imperialism competitive algorithm with application to retinal vessel segmentation. *Biocybernetics and Biomedical Engineering*.

[38] Sundaram R., Ks R., Jayaraman P., B V. (2019). Extraction of blood vessels in fundus images of retina through hybrid segmentation approach. *Mathematics*.

[39] Dash S., Verma S., Kavita (2021). A hybrid method to enhance thick and thin vessels for blood vessel segmentation. *Diagnostics*.

[40] Dagli Y., Choksi S., Roy S., Peter J., Fernandes S., Eduardo Thomaz C., Viriri S. (2019). Prediction of two year survival among patients of non-small cell lung cancer. *Computer Aided Intervention and Diagnostics in Clinical and Medical Images*.

[41] Primitivo D., Alma R., Erik C. (2019). A hybrid method for blood vessel segmentation in images. *Biocybernetics and Biomedical Engineering*.

[42] Hashemzadeh M., Adlpour Azar B. (2019). Retinal blood vessel extraction employing effective image features and combination of supervised and unsupervised machine learning methods. *Artificial Intelligence in Medicine*.

[43] Anand V., Gupta S., Koundal D., Nayak S. R., Barsocchi P., Bhoi A. K. (Jan 2022). Modified U-net architecture for segmentation of skin lesion. *Sensors*.

[44] Khawaja A., Khan T. M., Naveed K., Naqvi S. S., Rehman N. U., Junaid Nawaz S. (2019). An improved retinal vessel segmentation framework using frangi filter coupled with the probabilistic patch based denoiser. *IEEE Access*.

[45] Wang B., Wang S., Qiu S., Wei W., Wang H., He H. (2021). CSU-net: a Context spatial U-net for accurate blood vessel segmentation in fundus images. *IEEE Journal of Biomedical and Health Informatics*.

[46] Chen C., Chuah J. H., Ali R., Wang Y. (2021). Retinal vessel segmentation using deep learning: a review. *IEEE Access*.

[47] Kriplani H., Patel B., Roy S., Peter J., Fernandes S., Eduardo Thomaz C., Viriri S. (2019). Prediction of chronic kidney diseases using deep artificial neural network technique. *Computer Aided Intervention and Diagnostics in Clinical and Medical Images*.

[48] Srinivasu P. N., SivaSai J. G., Ijaz M. F., Bhoi A. K., Kim W., Kang J. J. (2021). Classification of skin disease using deep learning neural networks with MobileNet V2 and LSTM. *Sensors*.

[49] Ijaz M. F., Attique M., Son Y. (2020). Data-driven cervical cancer prediction model with outlier detection and over-sampling methods. *Sensors*.

[50] Malarvel M., Nayak S. R. Region grow using fuzzy automated seed selection for weld defect segmentation in x-radiography image.

[51] Naga Srinivasu P., Ahmed S., Alhumam A., Bhoi Kumar A., Fazal Ijaz M. (2021). An AW-HARIS based automated segmentation of human liver using CT images. *Computers, Materials & Continua*.

[52] Vulli A., Srinivasu P. N., Sashank M. S. K., Shafi J., Choi J., Ijaz M. F. (2022). Fine-tuned DenseNet-169 for breast cancer metastasis prediction using FastAI and 1-cycle policy. *Sensors*.

[53] Rizwan I Haque I., Neubert J. (2020). Deep learning approaches to biomedical image segmentation. *Informatics in Medicine Unlocked*.

[54] Sood M., Verma S., Panchal V. K., Kavita fnm (2019). Optimal path planning using swarm intelligence based hybrid techniques. *Journal of Computational and Theoretical Nanoscience*.

[55] Huang S.-C., Cheng F.-C., Chiu Y.-S. (2013). Efficient contrast enhancement using adaptive Gamma correction with weighting distribution. *IEEE Transactions on Image Processing*.

[56] Rani S., Koundal D., Kavita fnm, Ijaz M. F., Elhoseny M., Alghamdi M. I. (2021). An optimized framework for WSN routing in the Context of industry 4.0. *Sensors*.

[57] Dash S., Jena U. R., Senapati M. R. (2018). Homomorphic normalization-based descriptors for texture classification. *Arabian Journal for Science and Engineering*.

[58] Gaur L., Singh G., Solanki A. (2021). Disposition of youth in predicting sustainable development goals using the neuro-fuzzy and random forest algorithms. *Hum. Cent. Comput. and Inf. Sci.*.

[59] Agrawal S., Panda R., Mishro P. K., Abraham A. (2022). A novel joint histogram equalization based image contrast enhancement. *Journal of King Saud University - Computer and Information Sciences*.

[60] Kaur M., Verma S., Kavita (2020). Flying ad-hoc network (FANET): challenges and routing protocols. *Journal of Computational and Theoretical Nanoscience*.

[61] Lin S. C. F., Wong C. Y., Jiang G. (2016). Intensity and edge based adaptive unsharp masking filter for color image enhancement. *Optik*.

[62] Sharma T., Verma S., Kavita (2017). Prediction of heart disease using Cleveland dataset: a machine learning approach. *Int. J. Rec. Res. Asp.*.

[63] Kwok N., Shi H. Design of unsharp masking filter kernel and gain using Particle Swarm Optimization.

[64] Ghosh G., Kavita fnm, Anand D. (2021). Secure surveillance systems using partial-regeneration-based non-dominated optimization and 5D-chaotic map. *Symmetry*.

[65] Hasler D., Suesstrunk S. E. (2003). Measuring colorfulness in natural images. *SPIE Proceedings*.

